# Effects of long-distance transportation on blood constituents and composition of the nasal microbiota in healthy donkeys

**DOI:** 10.1186/s12917-020-02563-5

**Published:** 2020-09-15

**Authors:** Fuwei Zhao, Guimiao Jiang, Chuanliang Ji, Zhiping Zhang, Weiping Gao, Peixiang Feng, Haijing Li, Min Li, Haibing Liu, Guiqin Liu, Humberto B. Magalhaes, Jianji Li

**Affiliations:** 1 College of Veterinary Medicine, Yangzhou University, 225009 Yangzhou, P.R. China; 2 National Engineering Research Center for Gelatin-based TCM, Dong-E E-Jiao Co., Ltd, 78 E-Jiao Street Donge County, Liaocheng, 252201 Shandong Province China; 3 Key Laboratory of Zoonosis of Liaoning Province, College of Animal Science & Veterinary Medicine, Shenyang Agricultural University, 110866 Shenyang, China; 4 The College of Animal Science and Veterinary Medicine, Henan Agricultural University, 450002 Zhengzhou, China; 5 College of Agronomy, Shandong Engineering Technology Research Center for Efficient Breeding and Ecological Feeding of Black Donkey, Liaocheng University, Shandong Donkey Industry Technology Collaborative Innovation Center, Liaocheng, China; 6 Department of Veterinary Surgery and Animal Reproduction, School of Veterinary Medicine and Animal Science, São Paulo State University (UNESP), Sp 18618-681 Botucatu, Brazil

**Keywords:** donkey, transport stress, 16S rRNA sequencing, nasal microbiota

## Abstract

**Background:**

This study aims to determine the effects of transportation on the nasal microbiota of healthy donkeys using 16S rRNA sequencing.

**Results:**

Deep nasal swabs and blood were sampled from 14 donkeys before and after 21 hours’ long-distance transportation. The values of the plasma hormone (cortisol (Cor), adrenocorticotrophic hormone (ACTH)), biochemical indicators (total protein (TP), albumin (ALB), creatinine (CREA), lactic dehydrogenase (LDH), aspartate transaminase (AST), creatine kinase (CK), blood urea (UREA), plasma glucose (GLU)) and blood routine indices (white blood cell (WBC), lymphocyte (LYM), neutrophil (NEU), red blood cell (RBC), hemoglobin (HGB)) were measured. 16S rRNA sequencing was used to assess the nasal microbiota, including alpha diversity, beta diversity, and phylogenetic structures. Results showed that levels of Cor, ACTH, and heat-shock protein 90 (HSP90) were significantly increased (*p* < 0.05) after long-distance transportation. Several biochemical indicators (AST, CK) and blood routine indices (Neu, RBC, and HGB) increased markedly (*p* < 0.05), but the LYM decreased significantly (*p* < 0.05). Nine families and eight genera had a mean relative abundance over 1%. The predominant phyla in nasal microbiota after and before transportation were *Proteobacteria*, *Firmicutes*, *Actinobacteria*, and *Bacteroidetes*. Transportation stress induced significant changes in terms of nasal microbiota structure compared with those before transportation based on principal coordinate analysis (PCoA) coupled with analysis of similarities (ANOSIM) (*p* < 0.05). Among these changes, a notably gain in *Proteobacteria* and loss in *Firmicutes* at the phylum level was observed.

**Conclusions:**

These results suggest transportation can cause stress to donkeys and change the richness and diversity of nasal microbiota. Further studies are required to understand the potential effect of these microbiota changes on the development of donkey respiratory diseases.

## Background

Bacterial microbiota is complex and plays a key role in human and animal health. The majority of studies have focused on the microbiota of the gastrointestinal tract through the analysis of intestinal contents or feces. Dysbiosis can be associated with a wide range of diseases in the gastrointestinal tract, including colitis [1] and transient diarrhea in foals [2]. However, recent research studies suggest that the nasal microbiota, which comprises a diverse and complex microbial population, is also crucial for host health and linked to increased risk of infection, contributing to the development of respiratory diseases [3]. Although other species are also being investigated, most nasal microbiota studies have been conducted in humans. For example, distinct histopathologic features of chronic rhinosinusitis are associated with the relative abundance of nasal microbiota phyla, specifically *Firmicutes* and *Bacteroidetes* [4]. Pulmonary, oral, and nasal microbiomes in horses are influenced by environmental conditions and are associated with health and mild-moderate equine asthma [5]. However, the populations of the nasal microbiota have not been assessed in donkeys.

Stress and exposure to respiratory pathogens could disrupt bacterial communities residing in the respiratory tract, reducing their capability to suppress pathogen colonization, overgrowth, or both [6]. Transportation exposes animals to various potential stress factors and causes severe stress. Previous studies found that respiratory problems, such as nasal discharge, coughing, inflammation/infection of the upper or lower respiratory tract, and pneumonia in the long haul transport of horses are very common, accounting for 27% of the incidence of transportation issues, which is not linked to a specific pathogen but rather a mixture of different bacterial species [7–9]. In recent years, transporting donkeys from traditional donkey-concentrated areas for fattening and breeding has become a major breeding model in China and has been accompanied by the increase in long-duration transportation, which also leads to high morbidity and mortality among donkeys during the recovery period. Moreover, respiratory diseases are found to be one of the main problems that gradually has becoming one of the key factors restricting the development of donkey breeding industry in China. Despite the high damage of donkey transportation, the effects of transportation on donkey microbiota regarding respiratory issues is poorly understood. For the first time, we used high-throughput pyrosequencing to evaluate the effects of transport on donkey nasal microbiota, which might give a new insight into the pathophysiology of diseases during recovery.

## Results

### Transportation of donkeys alters hormonal levels, haematobiochemical and hematological indices

The concentrations of plasma cortisol hormone (Cor), heat-shock protein 90 (HSP90), and adrenocorticotrophic hormone (ACTH) significantly differed before and immediately after transportation. Plasma ACTH, Cor, and HSP90 of the donkeys were significantly increased (*p* < 0.05) on the day of arrival compared with those on the day before transportation (Table. 1).

**Table 1:** Serum concentrations (mean ± SD) of stress hormones in healthy donkeys before and after transportation

Hormone traits	Before transport	95% CI	After transport	95% CI
Cor (ng/ml)	68.0 ± 3.00	62.7–71.7	92.3 ± 2.90*	87.7–96.9
ACTH (pg/ml)	163.8 ± 31.88	109.8–245.0	315.8 ± 27.9 **	255.0-351.0
HSP90 (ng/ml)	9.79 ± 1.62	7.12–11.9	13.7 ± 1.14*	12.4–14.5

*Cor* Cortisol; *ACTH* Adrenocorticotrophic; *HSP90 *Heat shock protein; *CI* Confidence interval. * denotes *p* < 0.05; ** denotes *p* < 0.01

NEU, RBC, and HGB levels significantly increased right after transport relative to their pre-transport levels (*p* < 0.05). WBC showed no significant increase after transport (*p >* 0.05). By contrast, LYM significantly decreased after transport (*p* < 0.05) (Table. 2). The concentrations of plasma TP, ALB, LDH, CREA, UREA, and GLU showed no differences before and after transport (*p >* 0.05). Plasma AST and CK levels were significantly higher after transport (*p* < 0.05) (Table. 3).

**Table 2:** Changes of blood routine indices levels before and after transport

Blood routine indices	Before transport	95% CI	After transport	95% CI
WBC (10^9^/L)	12.4 ± 3.41	9.71–17.1	13.2 ± 3.08	9.65–14.2
LYM (10^9^/L)	6.87 ± 2.68	6.19–8.56	5.31 ± 1.67 *	4.51–6.07
NEU (10^9^/L)	4.81 ± 0.34	4.39–5.06	6.55 ± 1.32*	5.40–7.59
RBC (10^9^/L)	6.13 ± 0.82	4.91–6.59	7.19 ± 0.6*	5.82–8.05
HGB (g/L)	106.1 ± 4.80	101.8-113.2	119.9 ± 5.94*	112.9-123.4

*WBC* White blood cell; *LYM* Lymphocyte; *NEU* Neutrophil; *RBC* Red blood cell; *HGB* Hemoglobin; *CI* Confidence interval. * denotes *p* < 0.05

**Table 3:** Changes of biochemical indicators before and after transport

Biochemical indicators	Before transport	95% CI	After transport	95% CI
TP (g/L)	48.5 ± 2.90	44.4–55.5	50.9 ± 3.55	46.4-59.0
ALB (g/L)	26.5 ± 1.36	23.4–29.4	27.9 ± 1.78	24.0-30.2
CREA (µmol/L)	110.0 ± 7.97	96.6-127.4	112.7 ± 8.43	93.3-120.4
LDH (IU/L)	200.4 ± 13.6	175.6-219.3	217.5 ± 34.0	163.1-295.0
AST (IU/L)	203.7 ± 19.3	172.0-243.1	250.9 ± 26.1*	143.4-394.2
CK (IU/L)	142.1 ± 26.6	93.1–191.0	248.5 ± 28.6**	187.0-310.0
UREA (mmol/L)	3.85 ± 0.58	2.76–5.03	4.28 ± 0.59	3.71-5.42
GLU (mmol/L)	5.37 ± 0.46	5.39–5.98	5.75 ± 0.52	4.59-6.57

*TP* Total protein, *ALB* Albumin, *CREA* Creatinine, *LDH *Lactic dehydrogenase, *AST* Aspartate transaminase, *CK *Creatine kinase, *UREA *Blood urea, *GLU *Serum glucose, *CI* Confidence interval. * denotes *p* < 0.05; ** denotes *p* < 0.01

### Sequencing quality data and alpha diversity analysis

**Fig. 1:**
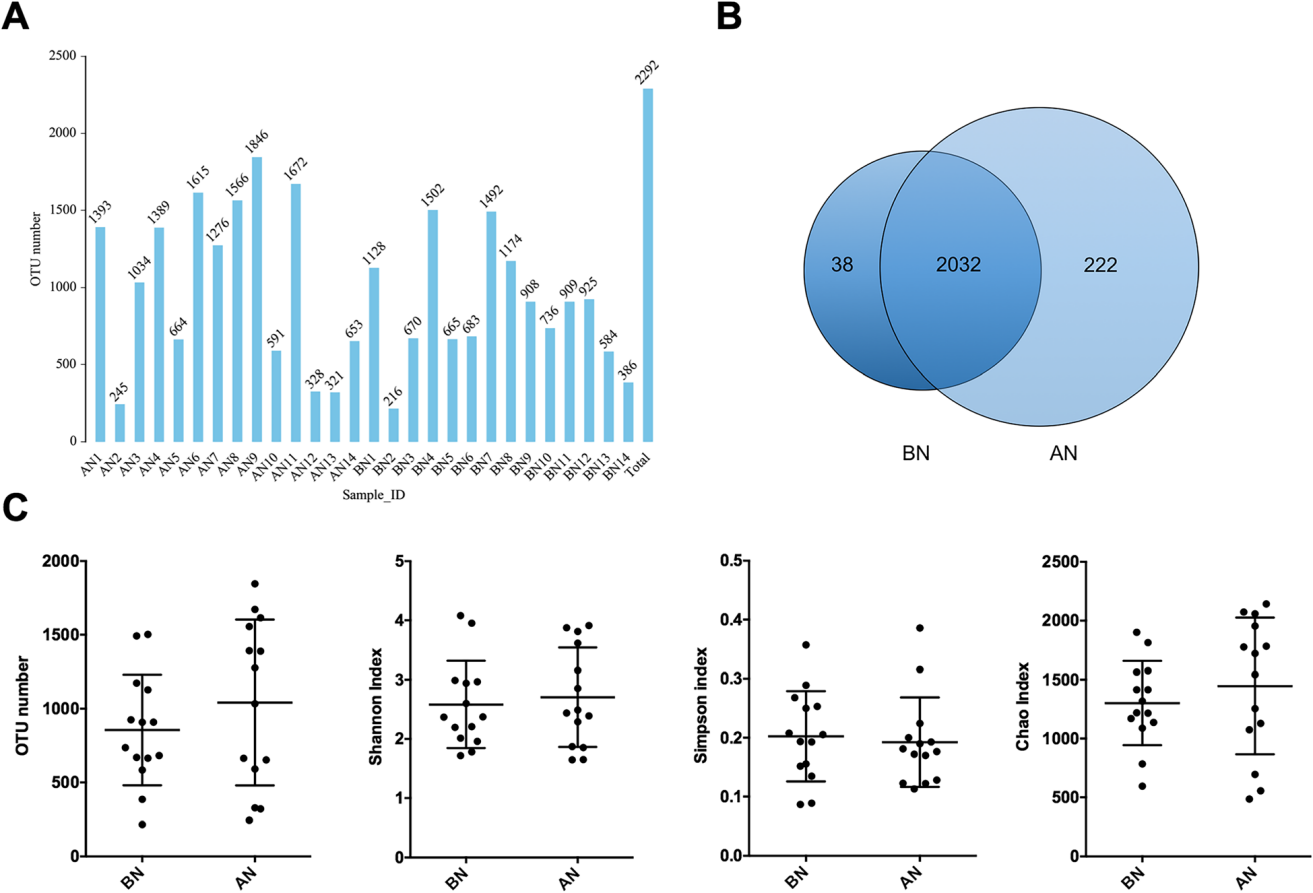
OUT analysis and alpha diversity indices of the nasal microbiota of healthy donkey (*n* = 14) before (BN) and after transport (AN). **a** The vertical axis (OTU Number) represents the final OTU number after taxonomic analysis. **b** Venn diagram of OTUs. The overlap section represents the shared OTUs between BN and AN group.**c **OTU number and Shannon, Simpson, and Chao indexes of nasal bacterial microbiota between BN and AN groups

The microbiota composition of the nasal swabs was assessed by sequencing the bacterial 16S rRNA V3 + V4 region. A total of 1,995,062 pairs of reads were obtained from the 28 samples. Double-end read splicing and filtering resulted in 1,735,325 clean tags, and each sample produced 41,913 clean tags on average. The tags were clustered into operational taxonomic units (OTUs) using QIIME (version 1.8.0) UCLUST software based on 97% sequence similarity. The number of OTUs in nasal swabs after transportation (AN1-14) slightly increased relative to that before transportation (BN1-14) (Fig. 1a). The Venn diagram of OTUs was illustrated (Fig. 1b), and the Chao, Simpson, and Shannon indexes were calculated (Fig. 1c). No significant differences in Chao, Simpson and Shannon indexes between before and after transportation were observed.

### Beta diversity analysis

**Fig. 2:**
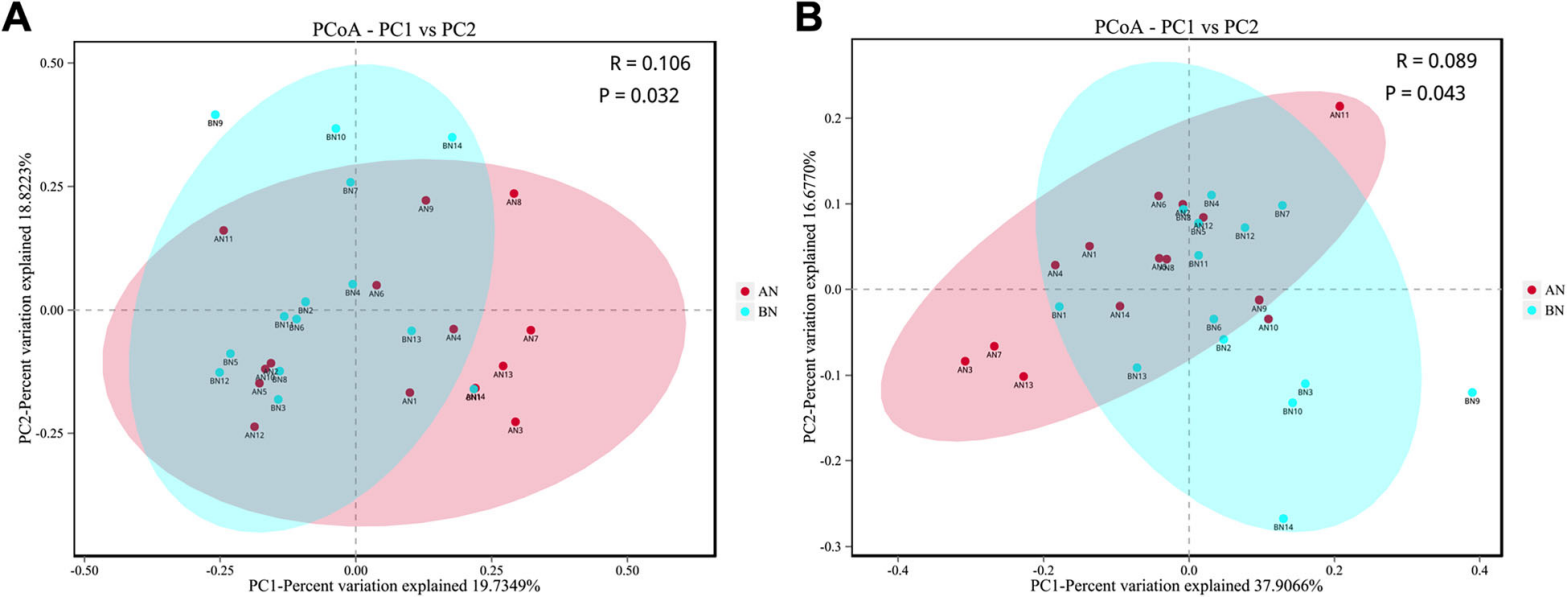
Principal coordinate analysis (PCoA) of the nasal microbiota of healthy donkeys (*n* = 14) before and after transport based on Bray-Curtis (**a**) and weighted UniFrac (**b**) algorithms. BN: Before transport, blue; AN after transport, red

Principal coordinate analysis (PCoA) between groups based on the Bray–Curtis (Fig. 2a) and weighted UniFrac (Fig. 2b) algorithms were performed to further explore the relationship among different bacterial communities before and after transportation. ANOSIM of weighted UniFrac and Bray–Curtis distances all showed that this clustering was significant (Bray–Curtis: *R* = 0.106, *p* = 0.032; weighted UniFrac: *R* = 0.089, *p* = 0.043).

### Phylogenetic analysis

**Fig. 3:**
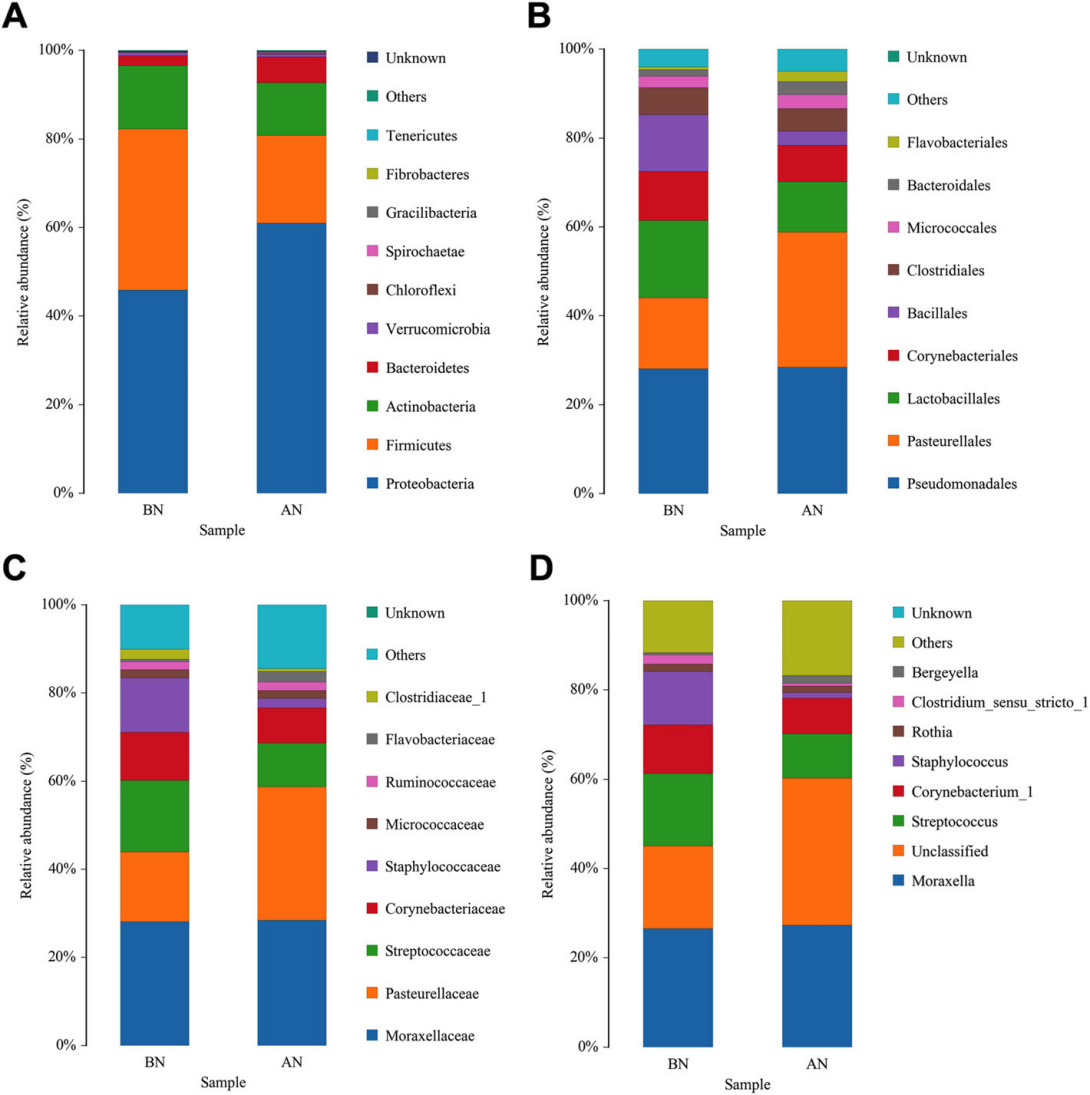
Relative abundance of predominant phyla (**a**), orders (**b**), families (**c**) and genera (**d**) in the nasal microbiota of healthy donkeys (n = 14) before (BN) and after (AN) long distance transport. Other: Bacterial taxa with ≤ 1% abundance, Unknown: Sequences which could not be classified

Ten phyla, nine orders, nine families, and eight genera had a mean relative abundance of over 1% (Fig. 3). The predominant phyla in each group were *Proteobacteria* (median: BN 45.9%, AN 61.0%), *Firmicutes* (median: BN 36.5%, AN 19.8%), *Actinobacteria* (median: BN 14.2%, AN 11.8%), and *Bacteroidetes* (median: BN 2.2%, AN 5.8%). Within *Proteobacteria*, the two most abundant families were *Moraxellaceae* (median: BN 28.1%, AN 28.4%) and *Pasteurellaceae* (median: BN 15.9%, AN 30.3%). *Streptococcaceae* (median: BN 16.2%, AN 9.9%) was the most abundant family within *Firmicutes*, followed by *Staphylococcaceae* (median: BN 12.4%, AN 2.1%) and *Ruminicoccaceae* (median: BN 1.8%, AN 1.9%). *Corynebacteriaceae* (median: BN 10.8%, AN 8.0%) was the most abundant family within *Actinobacteria*.

**Fig. 4:**
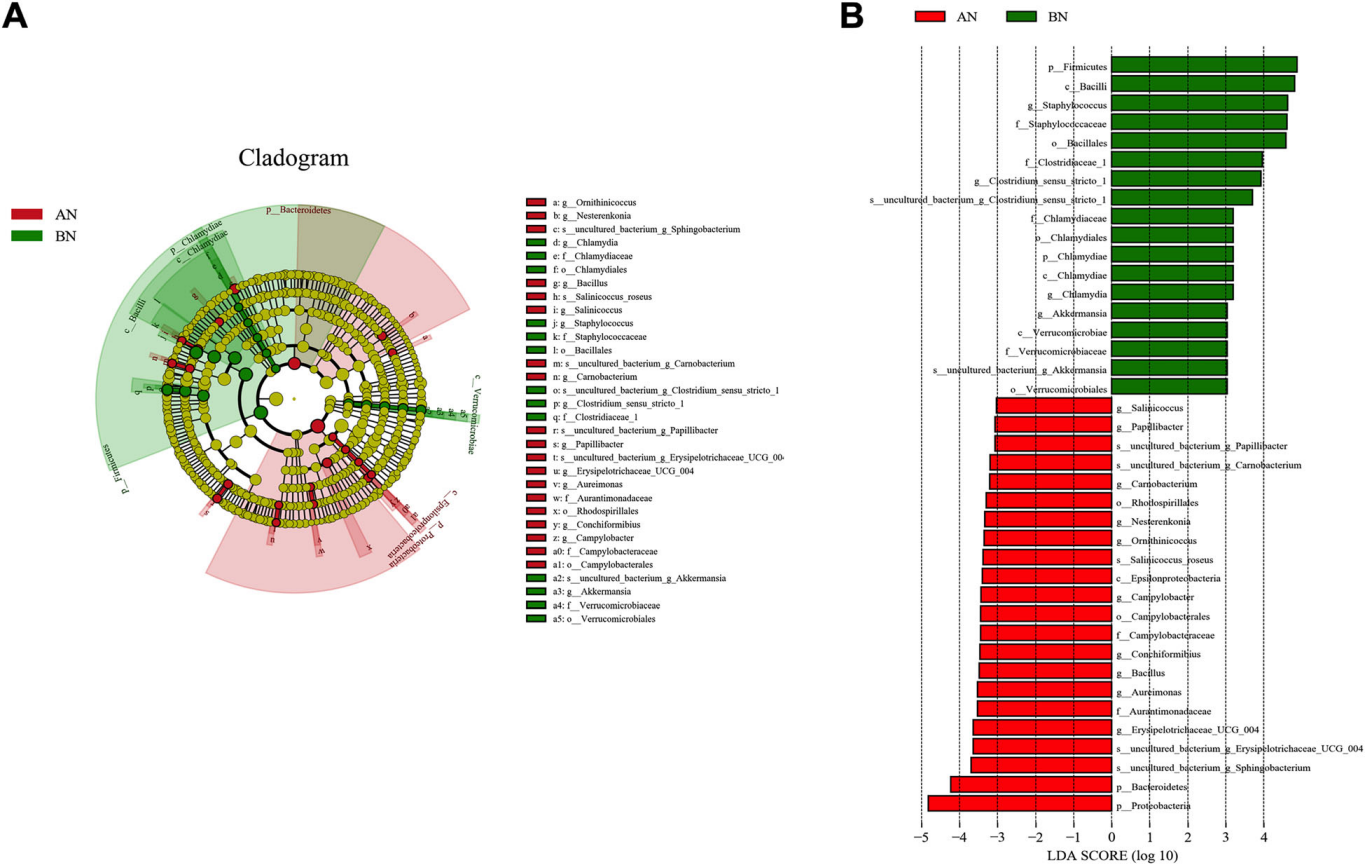
Linear discriminant effect size analysis (LEfSe) of the nasal microbiota of healthy donkeys (*n* = 14) before (BN) and after (AN) long distance transport. **a** Phylogenetic profile of specific bacterial taxa and predominant bacteria between the two groups. **b** Substantially high bacterial taxa at different taxonomic levels (p = phylum, c = class, o = order, f = family, g = genus, and s = species) represented in BN with (green) and AN (red) as indicated by LDA scores based on LEfSe. LDA score of 3.0 was used as a threshold for significance in LEfSe analyses

The linear discriminant analysis (LDA) effect size (LEfSe) method was used for the quantitative analysis of biomarkers in the microbiota among each group. The LDA score was set at 3.0, and different taxa with LDA threshold > 3.0 were considered significant biomarkers. The cladogram is shown in Fig. 4a, and the LDA score distribution map is shown in Fig. 4b. After transportation, donkey nasal microbiota showed an increase in the number of bacteria belonging to *Proteobacteria* and *Bacteroidetes* phyla and a decrease in bacteria belonging to the *Firmicutes* phylum.

## Discussion

The nasal mucous membranes are the first line of defense and can harbor important microorganisms that can be pathogenic under certain circumstances. The composition of the nasal microbiota has been shown to affect the severity of respiratory diseases [10]. For the first time, this study used the 16S rRNA sequencing technique to evaluate nasal microbiota composition changes in donkeys under transport stress.

ACTH and Cor levels increase under stress to deal with changes in the external environment. These hormones are important indexes in the stress reaction of animals, including beef cattle, piglets, chicken, and horses. During stressful situations, such as transportation, the ACTH and Cor contents in plasma variably increase [11, 12]. HSP90 is an important stress protein in organisms because it is rapidly activated and synthesized during stress reaction [13]. In this study, the ACTH, Cor, and HSP90 levels significantly increased after transport, and this finding is in agreement with other studies worldwide. For example, ACTH and Cor levels respectively increased to 4.9-fold and 1.8-fold higher than baseline after beef cattle transportation [14]. Similar to the above study, plasma Cor concentrations in transported horses greatly increased [11]. Therefore, we speculated that environmental disturbances (i.e., cold weather, over crowded, bumpy transportation) behave as stress factor, triggering a stress response in donkeys.

AST is an important indicator in liver function examination and CK is an indication of muscular activity [15, 16]. In this study, the AST and CK concentrations significantly increased, which is similar to previous studies [17–19]. Such increases of AST and CK might be due to liver damage or muscle breakdown caused by some physical stress, such as vehicle bumps, donkeys lying down, shaking, and excessive fatigue during transportation. Changes in leucocyte aggregation and dispersion induced by glucocorticoids may be the reason that damages the immune system, leading to an increase in total WBC count and but a decrease in LYM [20–22]. Consistent with other studies, our result showed that the total number of WBC and NEU increased, but LYM decreased, indicating that these changes are a response of donkey’s immune system to transport stress and possibly due to the elevated Cor or ACTH level during and/or after the transportation.

We hypothesized that nasal microbiota diversity and richness would be significantly altered after transportation. However, these aspects were unaffected according to the alpha diversity analysis in this study. Although increases were not statistically significant, OTU number and Chao and Shannon indexes were all higher after transport compared with those before transport. This finding indicates that donkeys after transportation tended to have an enriched nasal microbiota. Furthermore, the beta diversity was measured using the phylogeny-based weighted UniFrac distances. PCoA coupled with ANOSIM analysis showed significant differences in nasal microbiota before and after transportation, suggesting variations in microbial structure over time. This finding is consistent with the study that also demonstrated the shift of nasopharyngeal microbiota structure and significant increases in the observed OTU and Shannon diversity index following transportation of beef cattle to the feedlot [23].

Nasal microbiome is altered during transportation, and the changes in the microbial structure were mostly driven by the taxa of different relative abundance observed in LEfSe analysis. The most evident alteration is a notably gain in *Proteobacteria* and loss in *Firmicutes* at the phylum level after transport. *Proteobacteria* and *Firmicutes* were detected as the predominant phyla in the respiratory microbiota of horses [5]. *Proteobacteria* was associated with increased inflammation and respiratory tract diseases [4]. Noticeably, the proportion of the family *Pasteurellaceae* was partly responsible for the high amount of *Proteobacteria*, which demonstrated an evident increase (median: BN 15.9% to AN 30.3%). *Pasteurellaceae* is an early, opportunistic invader when pulmonary clearance mechanisms are compromised [24]. Previous studies indicated the high abundance of *Pasteurellaceae* in the nasal microbiome of horses immediately after transportation [7, 8, 25]. In addition, this family was increased in dogs with nasal neoplasia [26], pulmonary communities of asthmatic horses [27], and nasal microbiota of pigs from farms with Glässer’s disease [10]. We also observed high relative abundances of *Campylobacter* belonging to the phylum *Proteobacteria*. Members of the *Campylobacter* genus naturally inhabit birds, humans, horses, and other mammals and colonize numerous sites, including the intestinal tract, lungs, oral cavity, or blood [28]. These members inhabit the human anterior nares but not as a major colonizer [29]. In the past decade, an increasing number of *Campylobacter* species have been recognized as important pathogens in humans and animals, such as inflammatory bowel diseases (IBD), bacteremia, and lung infections [30]. These findings suggested that transportation might resulted in the prevalence of such opportunistic pathogens in the nares of donkeys and may become part of the disease process after transportation.

Based on findings from LEfSe, another evident change is the decreased abundance of *Firmicutes* (dominated by *Clostridia*) after transportation. The presence of family *Clostridiaceae* (class *Clostridia*, order *Clostridiales*), which is relatively abundant in the donkey nasal swab samples before transportation, was also reported in the nasopharynx of healthy dairy cattle [31], piglets [10], and dogs [26]. *Clostridiales* microbiota could induce the production of interleukin 22, a cytokine responsible for maintaining mucosal integrity, limiting Th2 cytokine production and response, and promoting immunity against bacteria in the intestine and lungs [32]. A decrease in the abundance of *Clostridiales* was observed in horses after transportation and affected with acute colitis [1, 33], foals with diarrhea [34], and pigs from farms with Glässer’s disease [10]. This finding indicates a potential link of this order to gastrointestinal and respiratory diseases. After transportation, another significant depletion in relative abundance of taxa is *Akkermansia*, which was found in 90% of horses studied, including gastrointestinal and upper and lower respiratory tracts [5, 35]. Gastrointestinal *Akkermansia* is a mucolytic bacterium that strengthens enterocyte monolayer integrity through adhesion to the intestinal epithelium and activates immune homeostasis, increasing host expression of antimicrobial peptides [36]. Previous studies showed the association of decreased relative abundance of *Akkermansia* with IBD [37, 38]. A recent study found that this bacterium was depleted in relative abundance in the nasopharynx microbial communities of children with a prior history of sinusitis [39]. However, the role of nasopharynx *Akkermansia* in the airway mucosal surface with the same mechanisms as the gut remains undetermined. Given that stress-induced impairment of the integrity of the intestinal epithelium reduces the efficacy of the innate protective mechanisms and may increase the potential for intestinal inflammation [40], we speculated that as a known stressor, transportation could disturb the integrity of donkey nasal mucosa and inflammatory homeostasis. Moreover, the decreased relative abundance of *Clostridiales* and *Akkermansia* may contribute to the modifications of mucosal integrity, thus increasing the risk of infection by other pathogens and provoking an inflammatory response.

This study had several limitations, including limited sample size, lack of a control group and inevitable environmental factors. The number of donkeys was limited because we had to select the donkeys with similar sex, age, weight, and transportation experience. Additionally, a limitation to our study may be the absence of a control group that stays on the farm before transportation to show that the observed changes in the microbiota are due to transport, rather than change over time. Moreover, environmental factors can partially influence nasal microbiota composition [41]. Previous studies documented the variation in nasopharyngeal microbiota of beef cattle and horses when moved to a new environment [23, 27]. In this study, the accumulation of faecal material within the transport vehicle may be an unavoidable contamination source and result in increased inhalation of enteric organisms. Increased numbers of airborne bacteria were reported within the transportation vehicle, but the bacterial species present were not recorded [7]. All the above bacterial contamination in transport vehicle may be able to multiply within nasal cavity and affect the composition of nasal microbiota. Therefore, we took measures to minimize the effects of these factors on the experiment; for example, collecting nasal swab samples immediately after arrival. However, controlling all environmental variables, such as environmental changes in carriage and contact among donkeys, is impossible. Another point we need to consider is that because most of the Chinese farmers are used to purchasing donkeys during winter for breeding in the following spring, long-distance transportation is mainly concentrated in winter. Our study was performed in a dry and cold climatic condition, therefore, these results are not likely applicable to donkeys transported in other environmental parameters, such as long-distance transportation of donkeys occurred in hot, humid weather. Despite these limitations, this study provided a comprehensive analysis of the effects of transport stress on the nasal microbiota in healthy donkeys for the first time. Understanding nasal microbial community changes before and after transportation will advance the development of effective prevention and treatment protocols of respiratory diseases in donkey transportation management in China.

## Conclusions

Overall, transportation causes stress to donkeys and substantial changes in terms of nasal microbiota structure following arrival at the destination. Richness and diversity of the nasal microbiota are slightly increased immediately after arrival, among which a notably gain in *Proteobacteria* and loss in *Firmicutes* at the phylum level is observed. Considering the prominence of commensals within the *Pasteurellaceae* family after arrival, which may become part of the disease process after transportation, is necessary.

## Methods

### Animals and transportation

Dezhou donkeys are a large somatotype ass and unique indigenous breed in China. Fourteen male Dezhou donkeys, aged 10–12 months weighing (140.8 ± 5.2 kg, mean ± SD), were carefully selected from Inner Mongolia Dong-E Black Donkey Animal Husbandry Co., Ltd in Chifeng City, Inner Mongolia Province, China. These animals were transported to a private breeding farm (Dong-E E-Jiao Co., Ltd, Shandong Province, China), producing Dezhou donkeys. The animals that aimed to provide genetic materials (i.e., semen) as select breeders (i.e., Jackass) to be used either for breeding or as a germplasm reservoir (i.e., frozen semen), were raised and upheld in the breeding farm.

The donkeys were clinically healthy and were provided free access to water and feed comprising hay and commercial concentrates daily. All donkeys had no previous experience of road transport and were not treated with antibiotics within 1 month. The average environmental temperature and humidity during transportation were − 10 °C and 28%, respectively. The surrounding walls of the truck (13.4 m long and 5.6 m wide) were equipped with iron guardrails, and the floor was iron with extremely thin bedding materials. The truck did not have roof coverings, and the donkeys were thus exposed to different weather conditions. The transport started from Chifeng City in Inner Mongolia Province at 17:00 p.m. on January 7, 2018 and arrived at Dong-E City in Shandong Province at 14:00 p.m. on January 8, 2018, which represented a travel time of approximately 21 hours and a distance of 950 km. The routes were secondary roads and expressways. Hay was placed in haynets accessible to each donkey. Donkeys were offered water 3 times during transit (23:00 January 7 and 5:00, 11:00 January 8). Diet and water were unchanged before and after transportation, and all donkeys were stabilized with daily access to hay and water. The donkeys were housed in the same barn, without any contact with other animals. The same feeding methods and times were used before and after transportation. The fodder was transported from the original location, thereby minimizing the effects of environment and food on the experiment.

### Sample collection

Before transportation and upon arrival, blood and nasal samples were collected from each donkey in the same order and following methods within 2 hours. Briefly, 15 ml of the blood sample was collected from the jugular vein of each donkey and placed in separate vials (EDTA). Each vial contained 5 ml of the blood sample. The blood samples were placed on ice, immediately transferred to the laboratory for analysis, and centrifuged at 3000 g for 20 min at 4 °C. The supernatants were stored in microtubes at − 80 °C until analysis. All laboratory analyses were performed within 24 h. Nasal swabs were taken as Tara G. McDaneld described in his study [42]. Nasal swabs were collected from the upper nasal cavity of all donkeys using 15 cm nasal swabs. For sampling, the nose of the animal was wiped cleaned with a single-use towel if fecal material was present. The unguarded 15 cm nasal swab was then gently inserted into the nasal cavity at an approximate depth of 15 cm. The nasal swab was then rotated and removed. After collection of the sample, all swabs were placed in buffered peptone water with 12% glycerol and stored at − 80 °C.

### Hormonal analysis, Hematological evaluation and Haematobiochemical analysis

Cortisol hormone (Cor), heat-shock protein 90 (HSP90), and adrenocorticotrophic hormone (ACTH) were determined through an ELISA-based technique using the commercial kits of Enzyme-linked Biotechnology (Shanghai Enzyme-linked Biotechnology Co., Ltd. China).

Hematological indexes were evaluated with a blood cell analyzer (Mindary BC-5000Vet Blood Cell Analyzer). Commercial kits (Shenzhen Mindary Biomedical Electronics Co., Ltd. Shenzhen, China) supplied by MINDARY a with testing protocol for each selected hematological parameter were used.

The plasma samples were evaluated for plasma glucose, total protein (TP), glutamic oxaloacetic transaminase (AST), glucose (GLU), albumin (ALB), creatine kinase (CK), lactate dehydrogenase (LDH), creatinine (CREA), P (phosphorus) and TG (triglyceride) levels with a biochemical analyzer (Mindary 1800 Chemistry Analyzer, Shenzhen, China). Commercial kits (Shenzhen Mindary Biomedical Electronics Co., Ltd. Shenzhen, China) supplied by MINDARY with testing protocol for each selected biochemical parameter were used.

### DNA extraction and pyrosequencing

Total bacteria DNA was extracted from the nasal swabs stored at − 80 °C using the genomic DNA extraction kit (Tiangen Company, Beijing, China) according to the manufacturer’s protocol. The quality and concentration of the extracted DNA were measured using a NanoDrop spectrophotometer (ND-1000, NanoDrop Technologies, Wilmington, DE, United States). The V3 and V4 regions of the 16S rRNA gene were amplified by PCR (95 °C for 5 min, followed by 25 cycles of 95 °C for 30 s, 50 °C for 30 s, 72 °C for 40 s, and 72 °C for 7 min) using specific bacterial primers (F: 5′-ACTCCTACGGGAGGCAGCA-3′, R: 5′-GGACTACHVGGGTWTCTAAT-3′). Indexed adapters were added to the ends of the primers. The PCR products were mixed with the same volume of 2 × loading buffer and were subjected to 1.8% agarose gel electrophoresis for detection. Samples with a bright main band of approximately 450 bp were chosen and mixed in equidensity ratios. Then, the mixture of PCR products was purified using a GeneJET Gel Extraction Kit (Thermo Fisher Scientific, Waltham, MA, United States). Sequencing libraries were validated using an Agilent 2100 Bioanalyzer (Agilent Technologies, Palo Alto, CA, United States) and quantified with a Qubit 2.0 Fluorometer (Thermo Fisher). Finally, paired-end sequencing was conducted using an Illumina HiSeq 2500 platform (Illumina, San Diego, California, USA) at Biomarker Technologies Co., Ltd (Beijing, China).

### Bioinformatics and data analysis

The raw paired-end reads from the original DNA fragments were merged using FLASH32 and assigned to each sample according to the unique barcodes. QIIME (version 1.8.0) UCLUST software was used based on 97% sequence similarity, and the tags were clustered into operational taxonomic units (OTUs). Alpha diversity index was evaluated using Mothur software (version, v.1.30). The number of sequences contained in each sample was standardized to compare the richness among the samples. Analysis measures included Shannon, Chao, and Simpson indexes. For beta diversity analysis, principal coordinate analysis (PCoA) between groups based on the Bray–Curtis and weighted UniFrac algorithms were obtained using QIIME. The linear discriminant analysis (LDA)-effect size (LEfSe) method was used for the quantitative analysis of biomarkers in each group. LEfSe analysis, an LDA threshold > 3, the non-parametric factorial Kruskal–Wallis sum-rank test, and the unpaired Wilcoxon rank-sum test were performed to identify the most differently abundant taxa. The lower and upper limits of the 95% CI for a proportion were calculated using SPSS 25 software. *P* values were calculated by the two-tailed Student’s t-test using GraphPad Prism software (GraphPad Prism Software Inc., San Diego, California, USA), and a *P* value of < 0.05 was considered significant for all comparisons.

## Data Availability

The datasets used and/or analyzed during the current study are available from the corresponding author on reasonable request.

## Abbreviations

OTUs: Operational Taxonomic Units
LEfSe: Linear discriminant analysis (LDA)-effect size
PCoA: Principal coordinate analysis
HSP90: Heat-shock protein 90
ACTH: Adrenocorticotrophic hormone
